# Intrinsic Defects and H Doping in WO_3_

**DOI:** 10.1038/srep40882

**Published:** 2017-01-18

**Authors:** Jiajie Zhu, Maria Vasilopoulou, Dimitris Davazoglou, Stella Kennou, Alexander Chroneos, Udo Schwingenschlögl

**Affiliations:** 1 King Abdullah University of Science and Technology (KAUST), Physical Science and Engineering Division (PSE), Thuwal 23955-6900, Saudi Arabia; 2 Institute of Nanoscience and Nanotechnology (INN), National Center for Scientific Research Demokritos, 15310 Aghia Paraskevi, Athens, Greece; 3 Department of Chemical Engineering, University of Patras, 26504, Patras, Greece; 4 Department of Materials, Imperial College, London, SW7 2AZ, United Kingdom; 5 Faculty of Engineering, Environment and Computing, Coventry Universidsaseet, Coventry CV1 5FB, United Kingdom

## Abstract

WO_3_ is widely used as industrial catalyst. Intrinsic and/or extrinsic defects can tune the electronic properties and extend applications to gas sensors and optoelectonics. However, H doping is a challenge to WO_3_, the relevant mechanisms being hardly understood. In this context, we investigate intrinsic defects and H doping by density functional theory and experiments. Formation energies are calculated to determine the lowest energy defect states. O vacancies turn out to be stable in O-poor environment, in agreement with X-ray photoelectron spectroscopy, and O-H bond formation of H interstitial defects is predicted and confirmed by Fourier transform infrared spectroscopy.

Tungsten oxide (WO_3_) is widely used in industry, as catalyst and catalytic support^1^,^2^,^3^. Intrinsic and/or extrinsic defects can tune the compound’s behavior, in particular the electrical and optical properties, leading to electrochromic and gasochromic applications as well as to potential in areas such as smart windows, gas sensors and optoelectonics^4^,^5^,^6^,^7^. Stoichiometric WO_3_ is transparent and insulating with a band gap of 3.0 eV to 3.3 eV^8^,^9^, while presence of O vacancies results in optical absorption (blue color due to gap narrowing) and electrical conductivity^10^,^11^. In addition, the electronic properties, in particular the band gap, are found to be sensitive to the spatial arrangement of the W and O atoms^8^,^9^. H, due to its small size, is able to migrate in many inorganic compounds and can occupy interstitial sites without large structural expansion. It is able to induce intrinsic defects that provide free electrons^12^,^13^, modify the band gap^14^, interact with O vacancies^15^,^16^, and induce insulator-to-conductor transitions^17^. Despite much progress, H doping therefore remains a challenge to metal oxide semiconductors. On the other hand, little is known about its potential to endow semiconductors with novel electronic features.

Although the ground state of WO_3_ has a *γ*-monoclinic structure, the compound can also crystallize in other phases^18^. The electronic properties associated with the different structures have been investigated by density function theory, predicting that O vacancies realize a +2 charge state in the monoclinic and cubic phases^19^. The energy barrier for O vacancy migration turns out to be higher than 0.37 eV^20^. In the present work we use density functional theory to study the stability of various defects as well as their influence on the electronic structure of WO_3_. In addition, we report facile routes to preparing stoichiometric, O-deficient (WO_3−*x*_), and H-sufficient (H_*z*_WO_3−*x*_) tungsten oxide. We investigate the electronic and optical properties by Fourier transform infrared (FTIR) and ultraviolet-visible (UV-vis) absorption spectroscopy, combined with X-ray and ultraviolet photoelectron spectroscopy (XPS, UPS).

## Results

**Figure 1:**
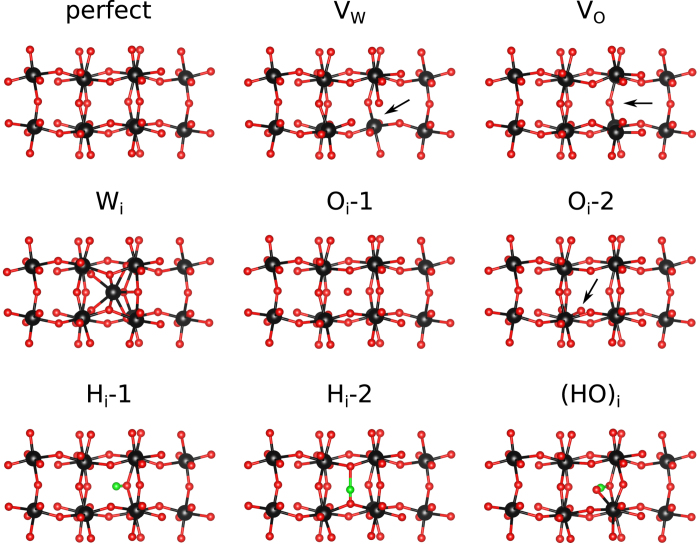
Structures: Perfect, with vacancies, and with interstitial defects, refer to the text for details. W, O, and H atoms are shown in black, red, and green color, respectively.

The lattice constants of *γ*-monoclinic WO_3_ are calculated to be *a* = 7.27 Å, *b* = 7.36 Å, and *c* = 7.54 Å, which agrees reasonably well with the experimental values (*a* = 7.31 Å, *b* = 7.54 Å, and *c* = 7.69 Å) and previous theoretical results (*a* = 7.39 Å, *b* = 7.64 Å, and *c* = 7.75 Å)^18^. The structural distortions induced by defects are illustrated in Fig. 1. We observe that the O atoms surrounding a W vacancy (V_w_) stay almost at their original positions, whereas nearby W atoms move towards an O vacancy (V_o_), which reduces the W-W distance from 4.18 Å (perfect structure) to 3.72 Å. Serveral locations along the face and body diagonals of the WO_6_ unit cell are tested for possible interstitial sites. We find that a W interstitial atom is stable only at the body center (W_i_) with a W-O bond length of 2.08 Å on average, which is significantly larger than in the perfect structure (1.93 Å). An O interstitial atom can be located at the body center (O_i_-1) or near a W atom (O_i_-2). The O-O distance of 2.12 Å in the O_i_-1 case shows that there is no O-O bonding (1.21 Å in an O_2_ molecule), whereas in the O_*i*_-2 case we obtain a W-O bond length of 1.92 Å. An H interstitial atom either can bond to a single O atom with a distance of 0.98 Å (H_i_-1) or it can be located at the face center with O-H distances of 1.01 Å and 1.67 Å (H_i_-2). Finally, an HO interstitial defect ((HO)_i_) is found to behave similarly to H_i_-2 with O-H distances of 1.02 Å and 1.50 Å.

**Figure 2:**
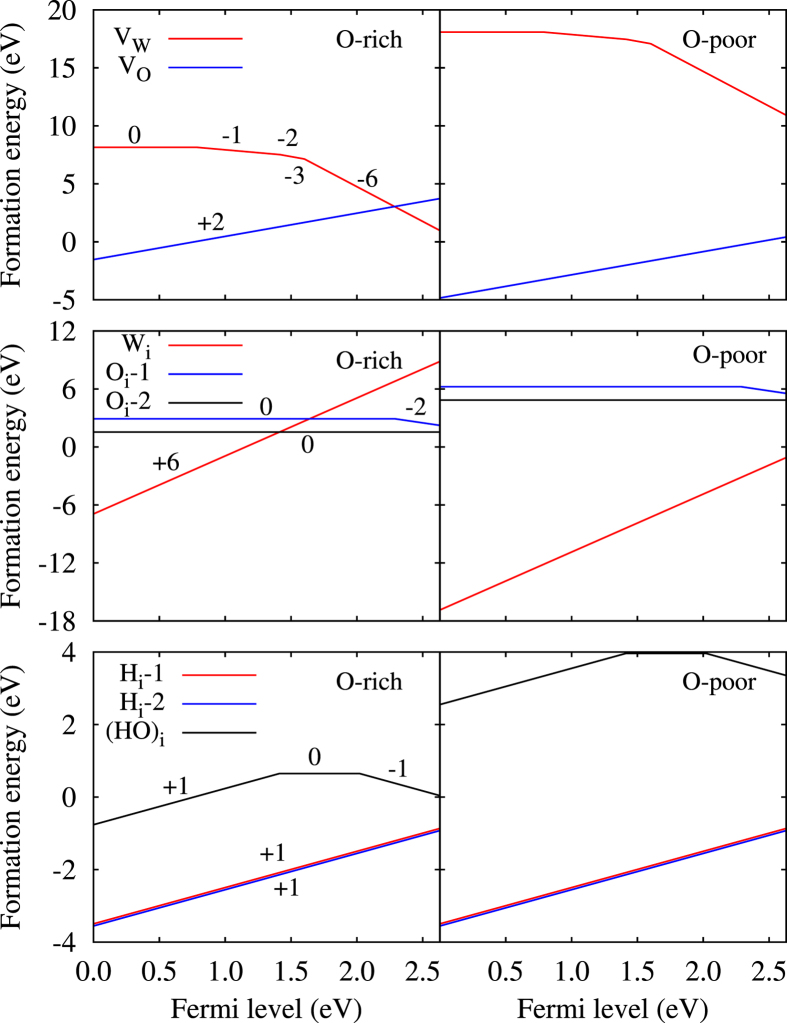
Formation energies of vacancies and interstitial defects for different charge states as functions of the Fermi level in the O-rich and O-poor limits. Zero Fermi level corresponds to the valence band maximum.

As expected, in the O-poor limit the formation energy of V_w_ is much higher than that of V_o_, which is negative for almost all values of the Fermi level, see Fig. 2. In the O-rich limit the situation changes qualitatively only for high values of the Fermi level. We observe that V_w_ is neutral when the Fermi level is near the conduction band minimum, while V_o_ realizes a +2 charge state, in agreement with previous theoretical results^19^. We note that our values for the formation energy of V_o_ are slightly lower than those of ref. 19, which is largely due to our improved treatment of the k-mesh. For V_w_ we find the thermodynamic transition levels *ε*(0/−1), *ε*(−1/−2), *ε*(−2/−3), and *ε*(−3/−6) at 0.79 eV, 1.42 eV, 1.59 eV, and 1.60 eV, respectively. The formation energy of O_i_-1 is higher than that of O_i_-2, since the former defect does not form chemical bonds. Due to bonding with six surrounding O atoms, W_i_ is only stable in the +6 charge state. The formation energy in the O-poor limit is (almost) strictly negative, while O_i_-1 and O_i_-2 show positive values. This order changes in the O-rich limit approximately when the Fermi level exceeds the middle of the band gap. We find for both H_i_-1 and H_i_-2 strictly negative formation energies because of O-H bonding, reflecting easy introduction of H in WO_3_. Introduction of H in the form of (HO)_i_ is possible only in O-rich environment due to the high formation energy of O_i_-2. For (HO)_i_ the thermodynamic transition levels *ε*(+1/0) and *ε*(0/−1) appear at 1.41 eV and 2.02 eV, respectively, see Fig. 2.

**Figure 3:**
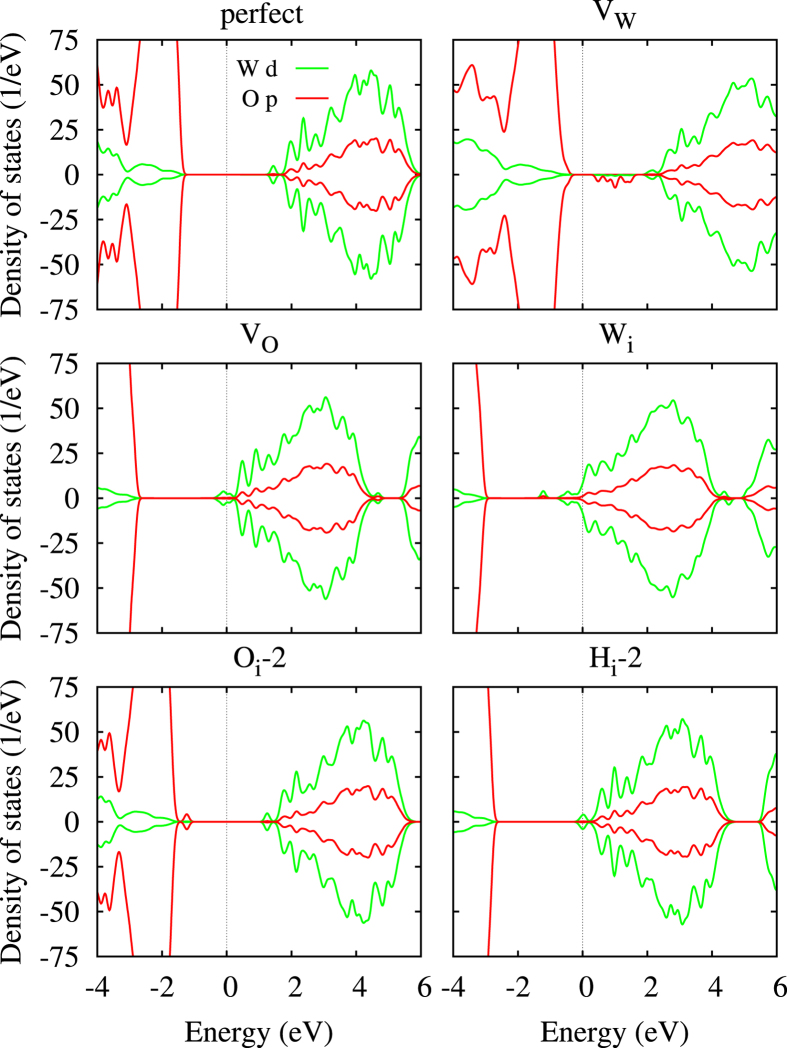
Densities of states of perfect WO_3_ and of selected defective supercells, calculated with a Gaussian smearing of 0.1 eV. Zero energy corresponds to the valence band maximum.

**Figure 4:**
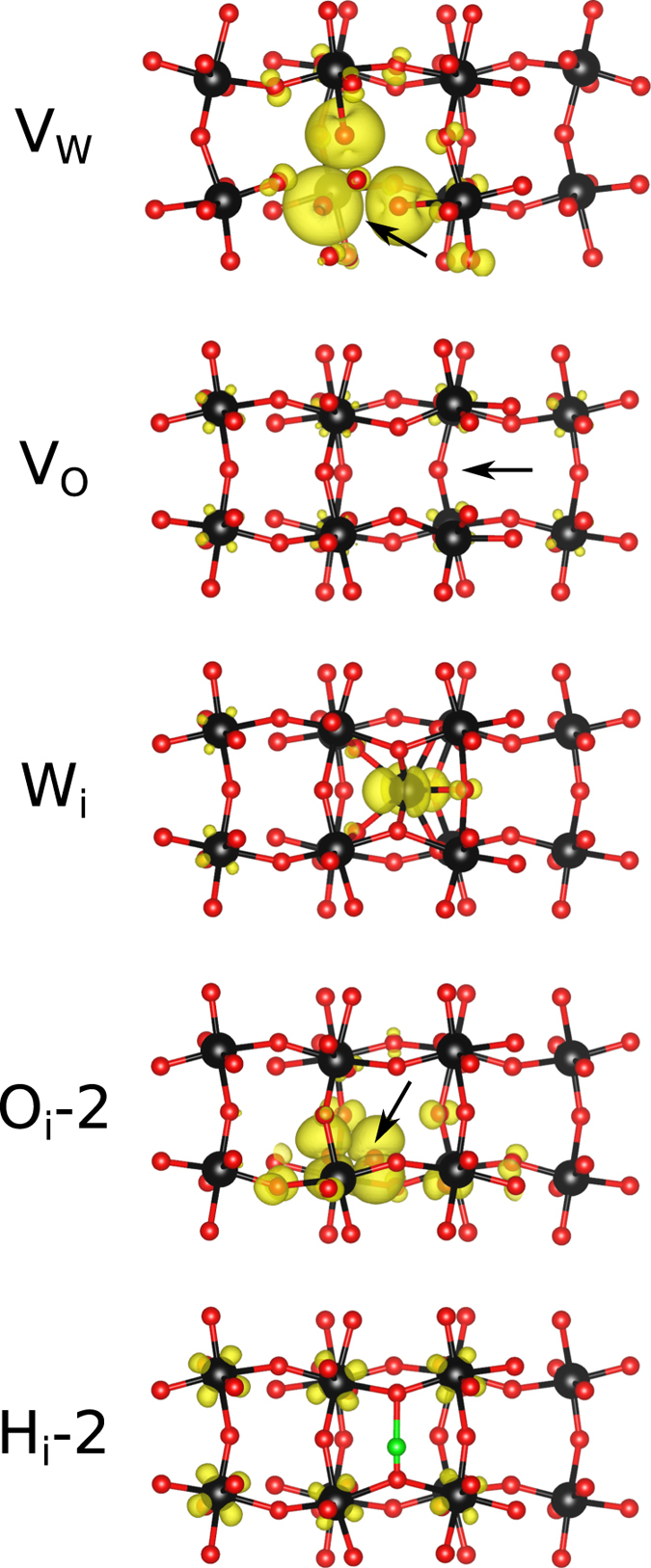
Charge densities of the in-gap states, see the text for details, plotted with isovalues of 0.006, 0.002, 0.006, 0.002, 0.001 electrons/bohr^3^ for V_w_, V_o_, W_i_, O_i_-2, and H_i_-2, respectively.

Figure 3 shows for perfect WO_3_ a (direct) band gap of 2.63 eV, in agreement with the experimental situation (2.6 eV to 3.2 eV)^18^ and a previous theoretical result (2.56 eV)^19^. The valence band maximum is almost purely due to the O 2*p* states and the conduction band minimum due to the W 5*d* states. For V_w_ and O_i_-2 the band gap is reduced to 0.70 eV and 2.23 eV, respectively, due to the presence of in-gap states, and it becomes indirect. For V_o_, W_i_, and H_i_-2 metallic characters are encountered, since the charge introduced by the defects enters the W 5*d* orbitals. Valence charge densities of the occupied (unoccupied in the case of V_w_) in-gap states (entire Brillouin zone) are shown in Fig. 4. For V_w_ they are located on three of the six neighbouring O atoms (reflecting pronounced charge ordering), while for V_o_ we obtain an almost uniform distribution over all W atoms in the supercell (in agreement with ref. 19). In the cases of W_i_ and O_i_-2, on the other hand, they are largely confined to the interstitial atoms and for H_i_-2 several W atoms around the defect are involved.

**Figure 5:**
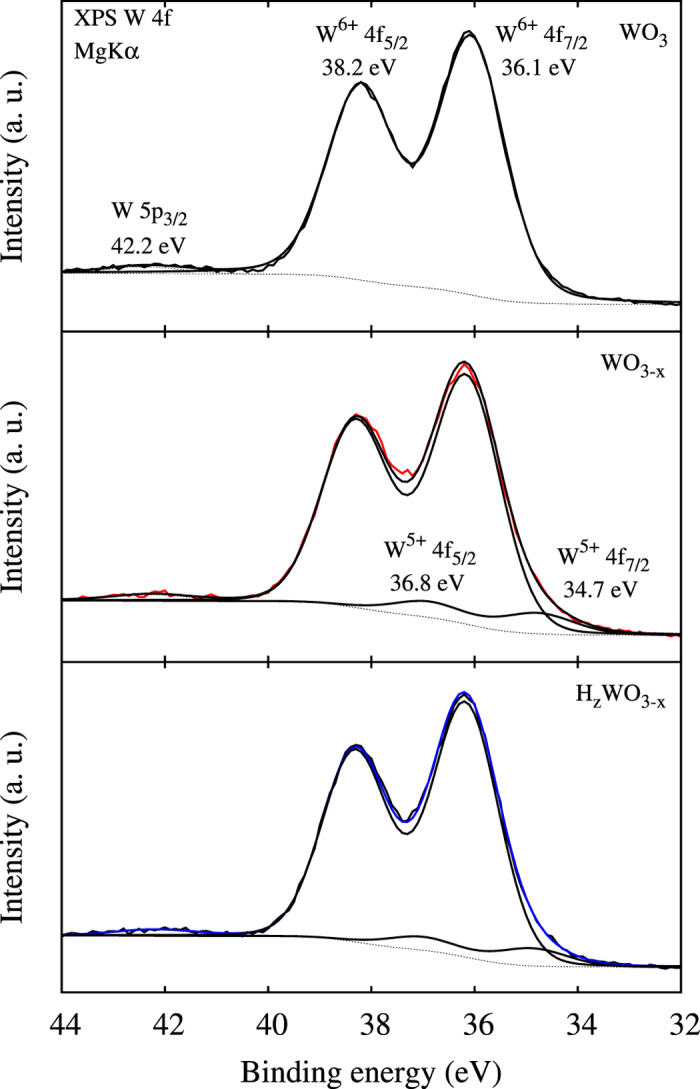
Surface W 4*f* XPS core level spectra and their deconvolutions.

Figure 5 shows W 4*f* XPS core level spectra obtained for films prepared in different environments. For WO_3_ the spectrum is deconvoluted in two peaks with weight ratio 4:3, nearly equal width of 1.7 eV, and binding energies of 36.1 ± 0.1 eV for W 4 f_7/2_ and 38.2 ± 0.1 eV for W 4 f_5/2_. The positions and shapes of these peaks agree with W in oxidation state +6, as expected for stoichiometric WO_3_^21^,^22^. For WO_3−*x*_ and H_*z*_WO_3−*x*_ the W 4*f* signal is broadened towards lower energy, reflecting the appearance of a new oxidation state lower than +6. Deconvolution of the spectrum demonstrates two distinct contributions: The W^6+^ doublet as found before and a minor doublet with weight ratio 4:3, width of 1.8 eV, and lower binding energies of 34.7 eV for W 4*f*_7/2_ and 36.8 eV for W 4*f*_5/2_. The new doublet represents W^+5^ ions^23^,^24^, which trace back to the presence of O vacancies.

**Figure 6:**
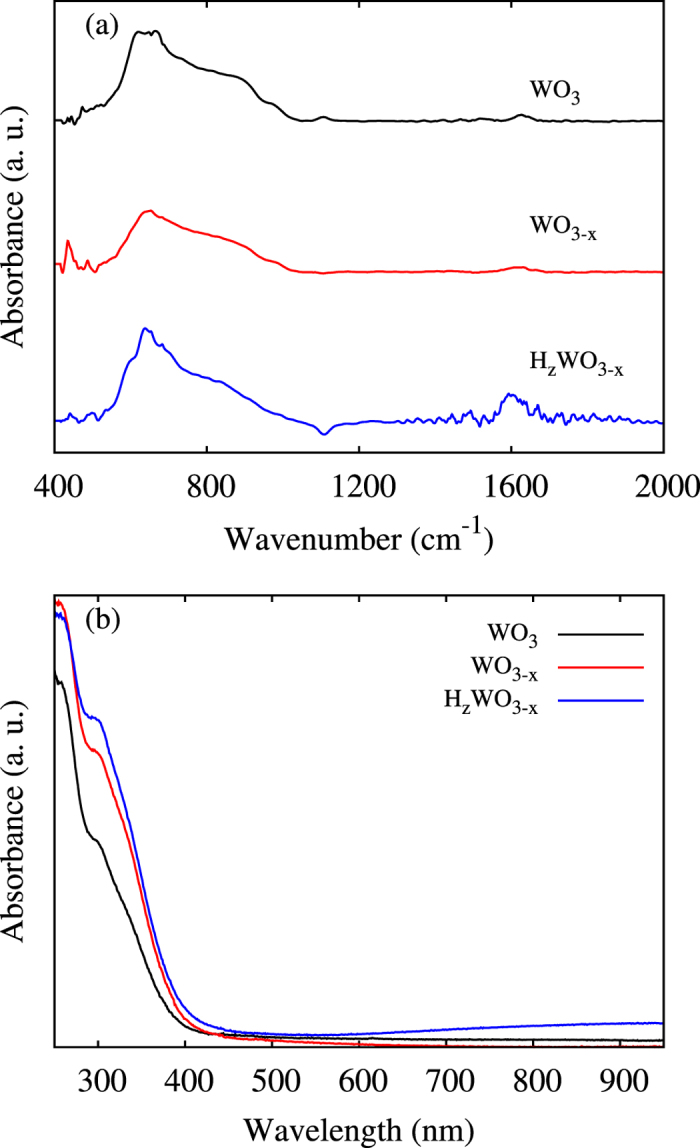
(**a**) FTIR and (**b**) UV-vis absorption spectra.

FTIR spectra, see Fig. 6(a), are measured to further elucidate the local structure changes induced by the different deposition environments. The spectra can be roughly divided into three regions: below 500 cm^−1^ with vibrations of the W-O bond, 500 cm^−1^ to 1100 cm^−1^ with vibrations of the W-O-W and O-W-O bonds, and above 1300 cm^−1^ with vibrations of the H-O-H bonds^25^,^26^. WO_3_ exhibits all these features with an additional shoulder near 910 cm^−1^, attributed to W=O bonds. The shoulder is suppressed for WO_3−*x*_ and H_*z*_WO_3−*x*_, reflecting the formation of O vacancies. Peaks observed around 1600 cm^−1^, mainly for H_*z*_WO_3−*x*_, indicate the presence of O-H bonds, in accordance with our theoretical results. Furthermore, the UV-vis adsorption spectra in Fig. 6(b) demonstrate energy gaps of 3.0 eV, 2.8 eV, and 2.75 eV for WO_3_, WO_3−*x*_, and H_*z*_WO_3−*x*_, respectively.

**Figure 7:**
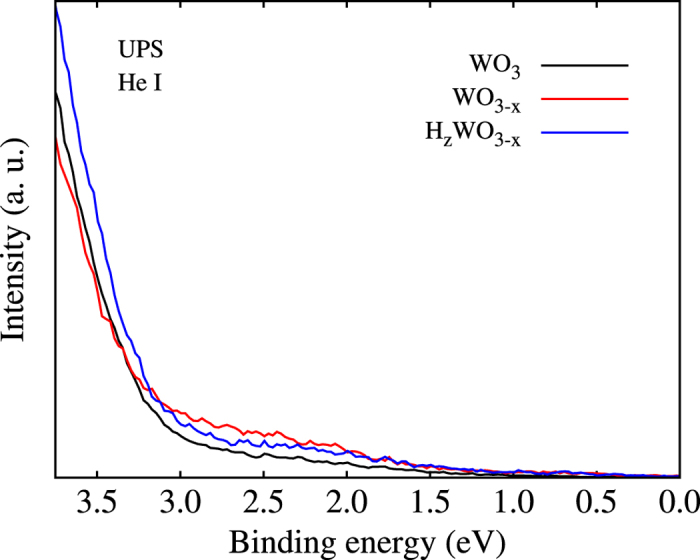
UPS spectra.

The UPS spectra in Fig. 7 show for WO_3_ the valence band maximum 2.9 ± 0.1 eV below the Fermi level, reflecting an n-type semiconductor, consistent with earlier experiments^23^. WO_3−*x*_ and H_*z*_WO_3−*x*_ exhibit similar valence band onsets. The main difference between the three oxides is related to the secondary electron cut-off region from which the work function can be estimated. We obtain values of 5.2 eV for WO_3_, WO_3−*x*_ and 5.6 eV for H_*z*_WO_3−*x*_. The work function of H_*z*_WO_3−*x*_ thus agrees with that of fresh tungsten oxide samples^27^,^28^, while the lower values of WO_3_ and WO_3−*x*_ can be explained by hygroscopic water uptake that occurs instantaneously in air and reduces the work function^29^. Since it is believed that water interacts with O atoms in the host^30^,^31^, H_*z*_WO_3−*x*_ probably maintains a high work function as the O atoms bonded with intercalated H are not available for this process. It recently has been demonstrated that annealing of WO_3_ in H_2_ atmosphere leads to O-deficient samples that show an order of magnitude enhancement in the photocurrent density^32^. Moreover, WO_2.72_ is a versatile and efficient catalyst for the hydrogenation of linear olefins, cyclic olefins, and aryl nitro groups^33^. In organic photovoltaics and organic light emitting diodes charge transport layers of O-deficient and H-sufficient WO_3_ can improve the performance of devices with forward architecture^34^.

Intrinsic defects and H doping in WO_3_ have been investigated by first-principles calculations, FTIR and UV-vis absorption spectroscopy, XPS, and UPS. The favorable defect states have been established. The prediction of low V_o_ formation energies in O-poor environment has been confirmed by the identification of a W^5+^ doublet by XPS. Multiple low energy peaks in the FTIR spectrum of H_*z*_WO_3−*x*_ have been attributed to vibrations of O-H bonds. UPS results on H_*z*_WO_3−*x*_ have demonstrated that the work function is enhanced efficiently by H intercalation.

## Methods

Density functional theory is employed based on the projector augmented wave method as implemented in the Vienna Ab-initio Simulation Package^35^. The generalized gradient approximation as proposed by Perdew, Burke and Ernzerhof^36^ (structure optimization) as well as the screened hybrid density functional proposed by Heyd, Scuseria, and Ernzerhof^37^ (formation energy and density of states) are used for the exchange correlation potential. The long range van der Waals interaction is taken into account by means of the DFT-D3 approach^38^. 2 × 2 × 2 supercells are used for all the defects to avoid artificial interaction because of the periodic boundary conditions. The cut-off energy for the plane wave basis is set to 500 eV and the energy tolerance for the iterative solution of the Kohn-Sham equations to 10^−6^ eV. All structures are relaxed until the residual forces on the atoms have declined to less than 0.03 eV/Å. We employ 2 × 2 × 2 k-meshes except for the hybrid density functional calculations of charged defects, for which Γ-point calculations are performed (to reduce the computational costs) and the total energy is corrected by comparison to the neutral counterparts (deviations ∼0.01 eV as compared to 2 × 2 × 2 k-meshes).

The defect formation energy is calculated as^39^


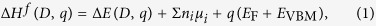


where Δ*E*(*D*, *q*) is the total energy difference between the perfect supercell and the supercell containing defect *D* in charge state *q*, *n*_*i*_ is the number of atoms of type *i* removed from the supercell, and *μ*_*i*_ is the corresponding chemical potential. Moreover, *E*_VBM_ and *E*_F_, respectively, are the valence band maximum and Fermi level (ranging from 0 eV to 2.63 eV, the size of the band gap). Stability of WO_3_ against byproducts and decompositions requires


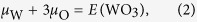



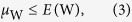



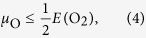



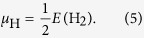


The O-rich and O-poor limits are given by the maximum and minimum values of *μ*_o_. Moreover, the thermodynamic transition level, for 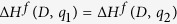, is defined as


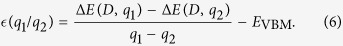


WO_3_ films are deposited in a system consisting of a stainless steel reactor with a W filament heated by an alternating current in order to vaporize its oxidized surface^21^. The chemical composition of the prepared oxide depends on the deposition environment: O_2_ (O-rich), N_2_ with traces of H_2_ (O-poor), or pure H_2_ (H-rich). The deposited WO_3_ films are characterized by FTIR absorption measurements using a Brooker spectrometer and UV-vis absorption measurements using a Perkin Elmer Lampda 40 UV/vis spectrophotometer. XPS measurements are conducted in ultra high vacuum (∼10^−10^ Torr) using a Leybold EA-11 analyzer and the unmonochromatized Mg K*α* line (photon energy 1253.6 eV) at 15 keV and 20 mA anode current. The instrument is calibrated for the Au 4*f*_7/2_ peak, giving a full width at half maximum of 1.3 eV. The stoichiometry is determined from the XPS W 4*f* and O 1*s* core level spectra. After Shirley background subtraction, the photoemission peaks are integrated by fitting the O 1*s* and W 4*f* spectra with asymmetric Gaussian-Lorentzian curves. The error is estimated to be ±10%. UPS spectra are recorded for 10 nm thick films deposited on Si substrate, using the same spectrometer as for the XPS measurements and the He I excitation line (photon energy 21.22 eV). The analyzer resolution is determined to be 0.16 eV from the width of the Au Fermi edge.

## Additional Information

**How to cite this article**: Zhu, J. *et al*. Intrinsic Defects and H Doping in WO_3_. *Sci. Rep.* **7**, 40882; doi: 10.1038/srep40882 (2017).

**Publisher's note:** Springer Nature remains neutral with regard to jurisdictional claims in published maps and institutional affiliations.
